# Cell and Tissue Therapy for the Treatment of Chronic Liver Disease

**DOI:** 10.1146/annurev-bioeng-112619-044026

**Published:** 2021-05-11

**Authors:** Yaron Bram, Duc-Huy T. Nguyen, Vikas Gupta, Jiwoon Park, Chanel Richardson, Vasuretha Chandar, Robert E. Schwartz

**Affiliations:** 1 Division of Gastroenterology and Hepatology, Department of Medicine, Weill Cornell Medicine, New York, NY 10065, USA;; 2 Department of Physiology, Biophysics and Systems Biology, Weill Cornell Medical College, New York, NY 10065, USA; 3 Department of Pharmacology, Weill Cornell Medical College, New York, NY 10065, USA

**Keywords:** tissue engineering, liver, cell therapy, stem cell biology

## Abstract

Liver disease is an important clinical problem, impacting 600 million people worldwide. It is the 11th-leading cause of death in the world. Despite constant improvement in treatment and diagnostics, the aging population and accumulated risk factors led to increased morbidity due to nonalcoholic fatty liver disease and steatohepatitis. Liver transplantation, first established in the 1960s, is the second-most-common solid organ transplantation and is the gold standard for the treatment of liver failure. However, less than 10% of the global need for liver transplantation is met at the current rates of transplantation due to the paucity of available organs. Cell- and tissue-based therapies present an alternative to organ transplantation. This review surveys the approaches and tools that have been developed, discusses the distinctive challenges that exist for cell- and tissue-based therapies, and examines the future directions of regenerative therapies for the treatment of liver disease.

## INTRODUCTION

Acute and chronic causes of liver disease are a growing clinical problem in the developed world, impacting over 30 million Americans and resulting in 750,000 hospitalizations and 36,000 deaths yearly, with similar numbers reported across western Europe (1). Unfortunately, unlike cardio-vascular diseases, the incidence of liver disease and its associated complications are increasing (2). Liver-associated diseases are responsible for approximately 2 million deaths per year worldwide. One million deaths are due to complications of cirrhosis and 1 million deaths are the result of viral hepatitis and hepatocellular carcinoma. Cirrhosis and liver cancer are the 11th- and 16th-most-common causes of global death, respectively in 2019. Together, they account for 3.5% of all deaths worldwide, which is a significant increase from 2000, when liver disease accounted for 3% of all deaths. Liver-related mortality is rising across the United States and Europe, as it is doing on a global scale (3). In addition to the increased risk of mortality, cirrhosis is one of the top 20 causes of disability-adjusted life-years and years of life lost, accounting for 1.6% and 2.1% of the worldwide burden (4). Cirrhosis-related mortality is rising and is expected to double over the next 20 years (5). The development of liver transplantation and its broad deployment cannot address the increasing needs for patients with liver disease. As a consequence, the fatality rate is over 20% for patients on the waiting list in the United States; this rate does not include all patients who would benefit from liver transplantation (6). Given the increasing burden that chronic liver disease places upon the health care system, there is a critical need for the development of new therapies.

The liver is the largest internal organ, performing a multitude of detoxification, immunologic, metabolic, and synthetic functions. The liver is unique in its capacity to heal after injury through regeneration rather than through scarring and reestablishment of the lost tissue mass through cellular hyperplasia (7, 8). Living donor transplantation takes advantage of this phenomenon, taking two-thirds of a donor liver for transplantation into a recipient, with mass restored in the ensuing months (9). Graft survival rates are better than rates for deceased donor transplantation, while recipient survival rates are similar to those for deceased donor transplantation, with survival rates at 90% for 1 year and 81% for 5 years (10). However, when injury processes occur too acutely (with loss of greater than two-thirds of liver mass) or occurs chronically (resulting in scarring), organ dysfunction occurs as a result of regeneration that is too slow to recover lost hepatic function and to allow for patient survival (11). The potential for liver regeneration and organ recovery is injury and patient specific and is hard to predict clinically, and consequently it is difficult to identify patients with liver injury who are likely to resolve and recover. Living donor transplantation is a forerunner of regenerative medicine therapies in the treatment of liver disease, relying on liver regeneration to expand the liver mass of both the host and recipient; however, similar to whole organ transplantation, its use is limited by organ paucity. As a consequence, alternatives to liver transplantation have been developed to support natural liver recovery or to act as a bridge to subsequent liver transplantation (12). These technologies, which include exchange/filtration systems such as molecular adsorbent recirculating system (MARS) and hepatocellular-based exchange/filtration systems such as extracorporeal liver assist device (ELAD), have been extensively studied but with minimal clinical success (13). As such, attention has turned to the development of cell- and tissue-based liver therapies for the treatment of chronic liver disease.

Given the different approaches that are currently in development for the treatment of liver disease, it will be helpful to place this work in the context of the varying clinical presentations of liver disease and discuss the physiological and metabolic abnormalities that characterize its dysfunction. Liver disease is evaluated and grouped according to its acuity and its resulting clinical complications. Acute liver failure refers to the development of severe liver injury with altered mental status (encephalopathy) and impaired synthetic function [international normalized ratio (INR) of ≥1.5] in a patient without cirrhosis or preexisting liver disease in a period of less than 26 weeks (11). INR is a globally standardized system of measurement of how long it takes for plasma to clot and is a good reference for whether a patient is at risk for bleeding or developing clots. Chronic liver disease or cirrhosis is the late stage of progressive hepatic fibrosis, which is characterized by distortion of the hepatic architecture and impaired synthetic function. Once cirrhosis develops, there is an escalated risk for the development of hepatocellular carcinoma and an increase in the resistance of flow in the portal vein, which can lead to portal hypertension. Commonly encountered complications of portal hypertension include variceal bleeding and ascites. Hepatic dysfunction combined with portal hypertension has impacts outside the liver affecting the brain (encephalopathy), heart (cirrhotic cardiomyopathy), kidneys (hepatorenal syndrome), and lungs (hepatopulmonary syndrome and portopulmonary syndrome).

Although originating in the liver, liver disease causes significant morbidity and mortality through systemic effects. Unfortunately, the only available treatment for acute and chronic liver disease is liver transplantation, which remains extremely limited. As a result, new and novel cell- and tissue-based therapies need to be developed to address this growing clinical need.

## CELLULAR COMPOSITION OF THE LIVER

**Figure 1:**
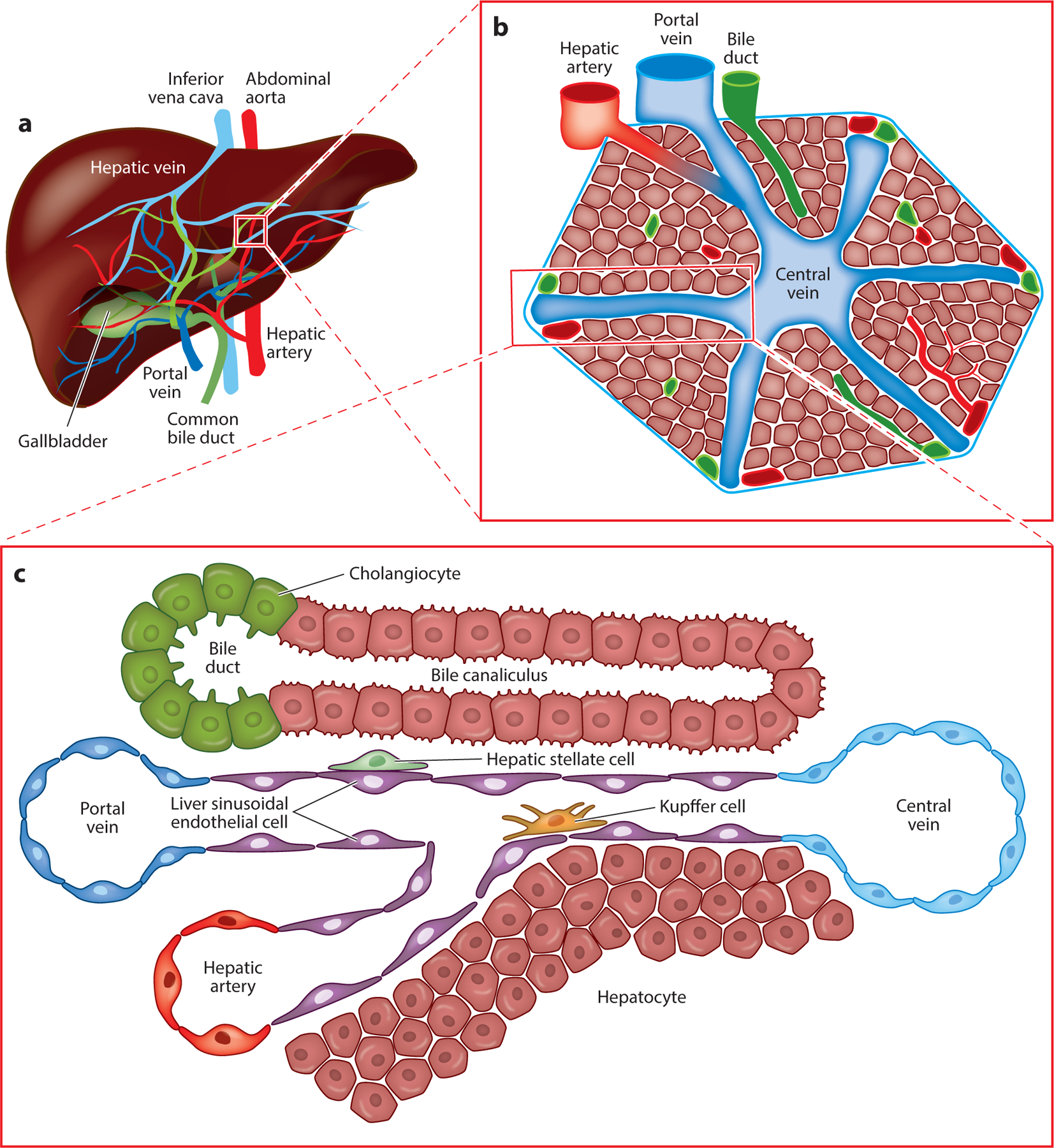
Architecture of the liver from the macro to the micro scale. (*a*) An example of the gross structure of the liver is shown. (*b*) When zoomed in, the lobular architecture of the liver, including the vascular and biliary components, is revealed. (*c*) Further examination reveals the cellular composition and architecture of the lobule.

To form hepatic tissue, multiple cell types must work in concert to carry out the many functions of the liver. These cell types are subdivided into the parenchymal cells that perform the chief biological processes of the liver and the nonparenchymal cells, which provide supportive roles for the parenchymal cells and overall organ function (Figure 1).

### Parenchymal Cells

Hepatocytes and cholangiocytes (also known as biliary epithelial cells) form the two main parenchymal cell types in the liver.

#### Hepatocytes.

In humans, the hepatocyte population composes ~80% of the liver’s mass and makes up ~60% of the liver by total cell number. One of the main functions of the liver is the secretion of various proteins into the blood, including clotting factors and albumin. Additionally, hepatocytes serve as a reservoir of glucose, storing it as a polymer (glycogen) that allows the hepatocytes to use gluconeogenesis to maintain homeostatic levels of blood glucose. Hepatocytes also create ~70% of the bile, which contains amphipathic bile acids, bilirubin (a breakdown product of heme in red blood cells), and fats (14). Besides these functions, they also perform a host of metabolic activities related to lipids (cholesterol, fatty acid, and lipoprotein synthesis, to name a few), and proteins (e.g., urea formation, amino acid synthesis). Lastly, hepatocytes modify and detoxify most compounds that enter the body through a vast array of P450 enzymes.

#### Cholangiocytes.

Cholangiocytes are either columnar or cuboidal in shape and line the ducts of the biliary tree. They compose about 3–5% of all liver cells in humans; however, they create an interconnected network estimated to be 1.25 miles in length (15). They interface with hepatocytes through the canals of Hering and merge into progressively larger ducts that culminate in the common bile duct, which expels bile into the gastrointestinal tract. Cholangiocytes produce ~30% of total bile flow and heavily modify the content of bicarbonate and other molecules.

### Nonparenchymal Cells

#### Endothelia.

Endothelia comprise a heterogenous mixture of cells that make up the majority of the nonparenchymal cells. Single-cell sequencing of human livers has depicted that endothelia of the sinusoids, central vein, portal vein, and lymphatics are distinct from one another. The liver sinusoidal endothelial cells (LSECs) are the best studied and known for their fenestrae, which allow blood and hepatocytes to interact, in addition to their other numerous functions (16, 17).

#### Mesenchymal cell types.

As with endothelia, there are a number of mesenchymal cell types within the liver whose heterogeneity we are just starting to understand. Hepatic stellate cells make up the majority of these cells, composing ~5–8% of total cells in the liver (18). They reside in the space between LSECs and hepatocytes and are known for their roles in vitamin A metabolism and fibrogenesis (19).

#### Kupffer and other immune populations.

Spaced throughout the liver is an impressive array of immune cells, including tissue resident macrophages (also known as Kupffer cells), natural killer cells, neutrophils, dendritic cells, B cells, and T cells (20). These cells serve a wide range of functions, including surveillance and inflammation.

## ARCHITECTURE OF THE LIVER

Divisions of the liver, known as lobules, govern the microanatomy of the liver (Figure 1). The organization of each cell type in this fashion allows the parenchymal cell types to carry out their various functions.

### Lobules

Blood flow into the liver lobule stems from two sources, the portal vein and the hepatic artery. The portal vein carries blood primarily from the digestive tract and mixes in a 2:1 ratio with arterial blood from the hepatic artery. This blood percolates from the portal tract through the hepatic sinusoids, where it interacts with hepatocytes before entering the central vein to return back to the heart. Bile secreted from hepatocytes enters a tiny channel known as a canaliculus that flows in the opposite direction of blood, from the central vein toward the portal tract. There it enters the smallest branches of the biliary tree and eventually enters the digestive tract.

### Zonation

While hepatocytes along the portal-central venous axis appear similar, it was discovered in the late 1970s that their position along this axis determined specialized functions (21, 22). Since then, a wealth of biology regarding hepatic zonation has been discovered. Traditionally, hepatocytes along the portal-central venous axis are divided into three zones: Those located adjacent to the portal tract constitute zone 1; those located adjacent to the central vein, zone 3; and those located in between, zone 2.

The best studied examples of hepatic zonation concern metabolism. Examples include glucose, nitrogen, and fatty acid metabolism. Zone 1 hepatocytes perform gluconeogenesis, urea synthesis from ammonia, and β-oxidation of fatty acids, whereas zone 3 hepatocytes break down glucose in the process of glycolysis, glutamine synthesis from residual ammonia by glutamine synthetase, and lipogenesis (23). With the advent of single-cell sequencing technologies, a more detailed map of zonation has emerged and has broadened our understanding of hepatic zonation (24–26).

## LIVER REGENERATION AND REPAIR: TARGETING NATURAL HEPATOCELLULAR SOURCES

Described in the ancient Greek myth of Prometheus, the liver has long been known to have a remarkable capacity to regenerate its mass, unlike other mammalian organs (7, 8). Many liver injury models have been employed to study this phenomenon, with the most common model being partial hepatectomy (27). In rodent models, removal of two-thirds of the liver mass results in compensatory hyperplasia and hypertrophy of the remaining lobes to restore original liver mass (28). This can be extended to a 90% resection in rats where glucose is provided during the recovery period (29). This proliferative response is conserved from fish all the way to humans and underlies what makes living donor liver transplantation possible.

**Figure 2:**
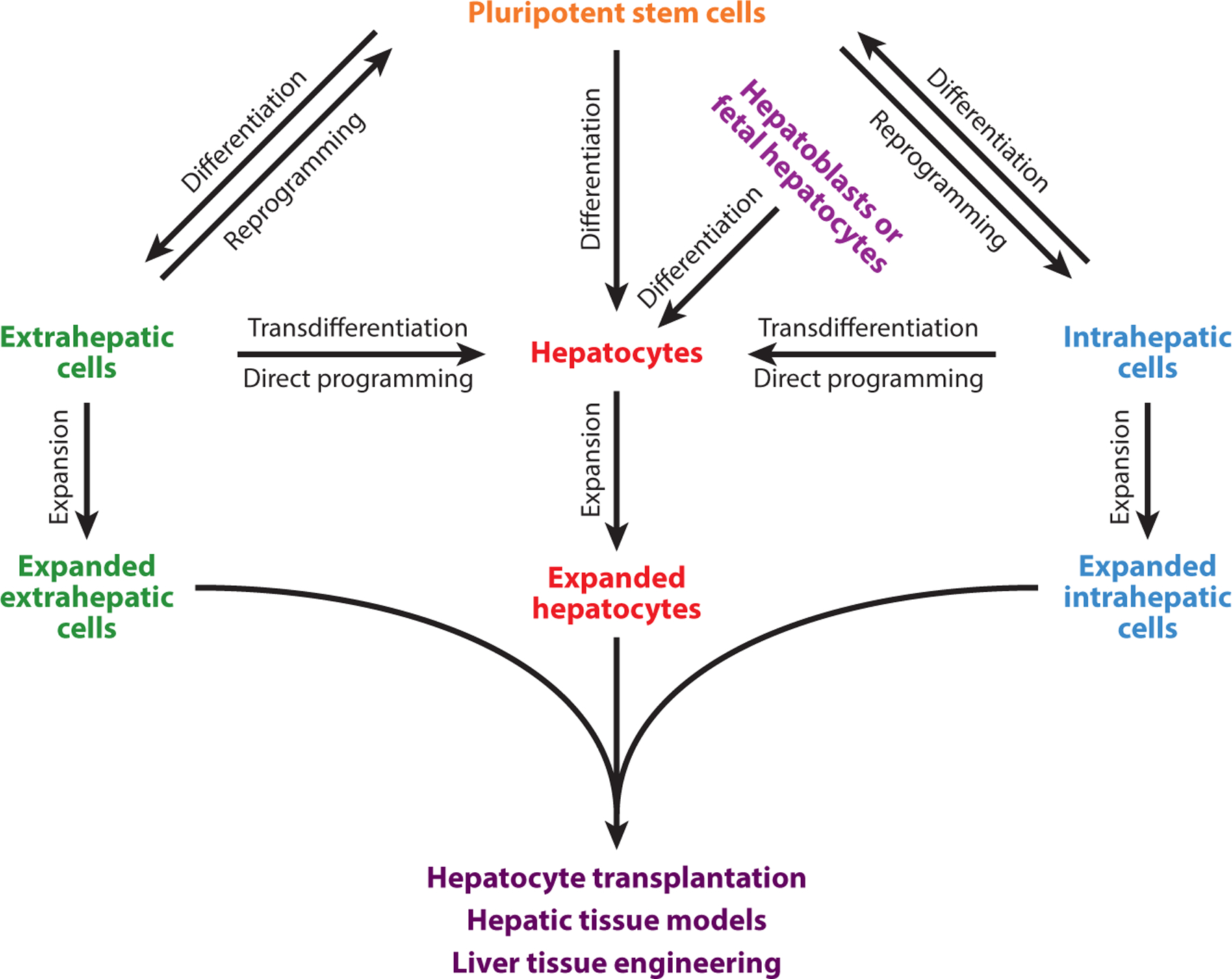
Different sources of hepatocytes for therapeutic and scientific applications. Many different cell types and sources can be differentiated, transdifferentiated, reprogrammed, or expanded to generate hepatocytes for clinical and scientific applications.

During the regenerative response, all cell types within the liver proliferate, including hepatocytes and cholangiocytes, which are the chief functional parenchymal cell types in the liver and bile ducts, respectively. While the liver contains a vast array of cell types, a major focus has been dedicated to expand these cells for regenerative medicine (Figure 2). Both of these cell types originate from a common developmental precursor known as the hepatoblast, which is characterized by the expression of both hepatocyte and cholangiocyte markers (30). As such, each of these cell populations has been interrogated with a wide variety of tools to determine their proliferative potency and ability to transdifferentiate into the other. Here, we review the current literature about the dynamic interrelationship of hepatocytes and cholangiocytes, including possible progenitor populations that serve as reservoirs for each.

### Hepatocytes as a Source for Hepatocytes

Given the robust proliferative response displayed from hepatocytes after partial hepatectomy, multiple groups have examined different subpopulations of hepatocytes to see whether one subpopulation has greater proliferative capacity. These studies have generally subdivided these populations by location along the liver lobule.

#### Periportal hepatocytes.

One of the oldest studies of hepatocyte renewal used a radioactive tracer (tritiated thymidine studies in rats), where periportal hepatocytes were labeled early and then appeared to migrate towards the central vein over 5 weeks (31). However, later genetic lineage tracing studies of periportal hepatocytes has shown that this is not likely to be the case during homeostasis. *Sox9*, which is primarily thought of as a marker of biliary epithelial cells, also has low-level expression within the single-cell layer of hepatocytes surrounding the portal vein in mice, so-called hybrid hepatocytes. Nine months after labeling *Sox9*^+^ periportal hepatocytes, there was no expansion of these labeled hepatocytes during homeostasis (32). Giving support to this idea, labeling murine periportal hepatocytes expressing *Mfsd2a*, which extends multiple hepatocyte layers out from the portal vein, showed that periportal hepatocytes decreased over 36 weeks (33). Intriguingly, both groups showed that periportal hepatocytes make significant contributions after liver injury.

#### Central venous versus distributed-based proliferation.

As periportal hepatocytes did not appear to be the primary driver of hepatocyte homeostasis, Wang & Nusse (34) examined the proliferative nature of pericentral hepatocytes. They found that hepatocytes marked by *Axin2*, a gene that is typically expressed in cells with active Wnt signaling, were present around the central vein. They incorporated nucleoside analogs at twice the rate of other hepatocytes, with genetically labeled progeny of *Axin2*^+^ cells giving rise to ~40% of new hepatocytes over one year (34). However, lineage tracing with a BAC-transgenic *Axin2:Cre*^*ERT2*^ did not recapitulate these results (35). Instead, pericentral hepatocytes remained around the central vein and did not show greater homeostatic proliferation compared with other hepatocytes. In agreement with these results are lineage tracing experiments with another Wnt-responsive gene, *Lgr5*, which was found to be coexpressed with *Axin2* in pericentral hepatocytes. Lineage tracing using *Lgr5* showed that ~50% of the clones remained as a single cell over 18 months, whereas <5% of cells gave rise to clones comprised of four cells. The same group also showed similar rates of nucleoside analog incorporation and Ki67 staining of hepatocytes within all three zones, challenging the notion that proliferation is regionalized (36). In agreement with this, multicolor clonal labeling of hepatocytes showed similar findings (37). As another approach, Lin and colleagues (38) utilized the telomerase (*Tert*) locus to find that *Tert* was expressed within 3–5% of all hepatocytes located throughout the liver lobule, which incorporated nucleoside analogs at a higher rate than *Tert*^−^ hepatocytes. Lineage tracing over one year showed that Tert^+^ hepatocytes expanded to cover ~30% of the liver area (38).

The discrepancy between these studies likely does not stem from one cause. In the case of *Axin2*, haploinsufficiency could be the culprit leading to differing results. Technically, differing Cre-LoxP labeling tools were utilized in each study. The expression pattern and strength of a Cre recombinase at a particular genetic locus combined with a given reporter line could lead to unanticipated outcomes if the tools are not properly characterized for specificity and leakiness. The labeling efficiencies of these reagents can also differ from each other, so it is possible that some experiments are not capturing enough hepatocytes within a given zone to truly understand organ-level proliferative dynamics. In some respects, it is also possible that all of these studies are correct in their own right, as it is known that all hepatocytes have the ability to proliferate when needed (8, 39). Further research will be needed to understand whether a highly proliferative subpopulation of hepatocytes exists within the normal liver or if proliferation is distributed throughout the lobule based on need.

### Hepatocytes as a Source for Cholangiocytes

Cholangiocytes, similar to hepatocytes, possess the capability to proliferate after major injury. After partial hepatectomy, cholangiocytes proliferate to expand the biliary tree to accommodate bile flow (40). Both hepatocytes and cholangiocytes are needed to fully restore a failing liver. However, several reports suggest that hepatocytes could be used as progenitor cells for cholangiocytes. Michalopoulos and colleagues (41) used bile duct ligation combined with the biliary toxin methylene dianiline (DAPM), which causes necrosis of the biliary epithelium in a rat model, and showed large swaths of bile ductules that emanated from hepatocytes. Generating chimeric mice using a fumarylacetoacetate hydrolase (FAH) model also resulted in similar findings. In this model, lack of FAH causes accumulation of toxic metabolites, which is prevented by the drug 2-(2-nitro-4-trifluoromethylbenzoyl)-1,3-cyclohexanedione (NTBC). Withdrawal of NTBC causes liver failure in adult animals, where therapies can be used to ameliorate this process. When rescued with FAH-expressing hepatocytes and withdrawal of NTBC, hepatocyte-derived ductules could be isolated and subsequently redifferentiate into hepatocytes upon serial transplantation (42). This type of transdifferentiation appears to be notch dependent, as overexpression of the notch intracellular domain within hepatocytes triggers expression of biliary markers (43). The most extreme example of such a process is exemplified in a mouse model of Alagille syndrome, in which hepatoblasts lack notch signaling and the biliary transcription factor *Onecut1* (*Hnf6*), leading to the absence of an intrahepatic biliary tree at birth. During postnatal growth, hepatocytes transdifferentiate to re-form the entire intrahepatic biliary tree (44). These studies demonstrate that hepatocytes appear to be able to compensate in certain conditions where cholangiocyte proliferation is impaired.

While many other studies have suggested that hepatocytes transdifferentiate or express biliary markers after exposure to injury (43, 45), the contribution of hepatocytes to the biliary lineage appears to be fairly limited when cholangiocytes retain proliferative capabilities. In multiple rodent models of liver injury, genetic labeling of virtually all hepatocytes shows that hepatocytes form more hepatocytes but do not transdifferentiate into cholangiocytes (46, 47). In an orthogonal approach, labeling all cholangiocytes, including cells within the canals of Hering, with *Hnf1b* or *Prom1* showed that cholangiocytes are the source of new cholangiocytes and reactive ductules (48, 49). While the contribution of hepatocytes to the biliary lineage might be minimal with cholangiocytes present, it does appear that, conceptually, hepatocytes could be utilized as a sole therapy given their ability to generate cholangiocytes when needed.

### Cholangiocytes as a Source for Hepatocytes

Similar to hepatocytes, cholangiocytes appear to retain an ability to transdifferentiate into hepatocytes if required. Two separate groups showed that ablating all hepatocytes in zebrafish larvae resulted in the conversion of cholangiocytes into hepatocytes (50, 51). In a similar type of experiment in mice, the regenerative potential of hepatocytes was impaired by deletion of β1 integrin (which causes hepatocyte necrosis and impaired response to growth factor signaling) or overexpression of p21 (which impairs hepatocyte proliferation). After injury and lineage tracing with *Krt19*, the authors found newly formed hepatocytes from cholangiocytes, with the authors estimating that ~25% of newly formed hepatocytes emanated from cholangiocytes (52). These models rely upon either extreme loss of hepatocytes or genetically impairing their ability to proliferate.

In more natural circumstances with injury, results have been somewhat mixed. *Krt19*-labeled cholangiocytes were seen to transdifferentiate into hepatocytes after 24 weeks of 3,5-diethoxycarbonyl-1,4-dihydrocollidine (DDC)- and thioacetamide-supplemented diet, accounting for ~10% of hepatocytes (53). Using *Opn* to label cholangiocytes, one group was able to see ~12% of the liver parenchyma occupied by transdifferentiated cells after 8 weeks of carbon tetra-chloride (CCL4) treatment (54). However, other approaches have shown no contribution. Labeling all hepatocytes with an adenovirus targeting only hepatocytes, it was shown in multiple injury models that hepatocytes gave rise to hepatocytes, suggesting no role of cholangiocyte derivation of hepatocytes (46, 47). Using *Krt19*, there was no transdifferentiation seen in multiple models of liver injury, including 3 weeks of DDC and 2 weeks of choline-deficient, ethionine-supplemented (CDE) diet (47). Using *Hnf1b*, which achieves an ~84% labeling efficiency of cholangiocytes, no transdifferentiation was seen through 8 months of DDC and 12 months in a Mdr2^−/−^ background. After 4 weeks of a CDE diet, there was low (1.5%) transdifferentiation of cholangiocytes into hepatocytes (48). Similar to hepatocyte lineage tracing, each promoter has differing specificities and rates of recombination. However, when all of the findings from these experiments are evaluated together, it does appear that cholangiocytes, similar to hepatocytes, could theoretically be used as a sole therapy given their plasticity.

### Progenitor Cells

The question of whether the liver contains a source of progenitor cells has been hotly debated and has generated significant controversy over the years. Emmanuel Farber in 1956 first described the histologic emergence of small, oval-shaped cells (oval cells) in a rat carcinogenesis model (55). Cells with similar characteristics were also observed in patient samples with genetic hemochromatosis, alcoholic liver disease, or chronic hepatitis C (56). Many studies have characterized these cells and initially defined them as liver endogenous progenitor cells, which can further differentiate to hepatocytes or cholangiocytes. Purified oval cells were shown to proliferate in vitro and differentiate into hepatocytes in the presence of epidermal growth factor (EGF) and hepatocyte growth factor (HGF) (57). Moreover, successful engraftment and function of oval cells transplanted in an FAH mouse model were demonstrated (58). More recent studies have used lineage tracing to identify the origin of these liver bipotent progenitors and showed (as described earlier) that primary hepatocytes exhibit phenotypic plasticity and can dedifferentiate, which promotes proliferation and restoration of the liver mass (42, 59). Multiple cellular markers identifying cell populations in the liver with clonogenic and multilineage potential have been reported over the years, such as EpCAM (60), CD13 (61), SOX9 (59), Foxl1 (62), MIC1–1C3 (63), CD24 (64), CD133 (65), and Lgr5 (66). In the last decade, Lgr5-positive cells have attracted much attention and were suggested to be an adult stem cell marker. LGR5 is a G protein–coupled receptor and is a member of the Wnt signaling pathway (67). First identified for its importance in intestinal epithelial renewal (68), Lgr5 expression in mouse liver was shown by Huch et al. (66) to be upregulated after tissue damage. Single Lgr5-positive cells were further isolated and expanded in vitro as organoids and retransplanted in an FAH mouse model. A CRISPR genetic screen in a mouse liver injury model showed that portal fibroblasts and periportal hepatocytes express Lgr5 after injury, which promotes hepatocyte-mediated regeneration but is not required during the ductular reaction, whereas YAP and mTORC1 signaling seems to be more dominant (69). Moreover, it was suggested that Lgr5 might also have a role in liver development, as it was shown to be expressed in the apex of the hepatoblast pool during mouse embryogenesis (70). Nonetheless, whether Lgr5 is expressed by truly bipotent progenitor cells during homeostasis and after liver damage and the correlation to other cellular markers are controversial. Further studies are required to determine the contribution of these and other proposed progenitor cells to liver regeneration and during liver injury, define their differentiation potential, and determine whether they can be used as a reliable cell source for transplantation or can be targeted as an endogenous therapy in a clinical setting.

## CELLULAR TRANSPLANTATION

### Primary Hepatocytes

Hepatocytes are the most abundant cell type in the liver, and since isolation protocols have been established, hepatocytes have been used in both in vitro and in vivo studies to investigate their function. Prior work examining hepatocyte transplantation (HT) into a mouse model with perpetual liver injury resulted in almost limitless hepatocyte proliferation and liver repopulation (39, 71). As a result, given this robust replicative capacity of hepatocytes in vivo, HT has been suggested as an alternative to orthotopic liver transplantation (OLT). Instead of involving transplantation of the whole liver as a graft, HT involves injecting freshly isolated or cryopreserved hepatocytes into the spleen or the portal vein; hepatocytes then migrate across the sinusoidal vessels to reach the liver parenchyma (72). HT can be performed using cells from rejected liver donor organs or even from partial liver resections, which would greatly expand the potential donor pool. There also have been efforts to directly reprogram hepatic myofibroblasts into hepatocytes through ectopic expression of the transcription factors (FOXA2, GATA4, HNF1A, and HNF4A), with the functionality of induced hepatocytes demonstrated both in vivo and in vitro (73, 74). The engrafted cells could augment hepatic function in patients with liver diseases. HT is theoretically less invasive and more cost-effective than OLT, as it can be performed repeatedly and with cells from either the same or different donors (75).

Since the first animal studies in 1976 and the first clinical attempt in 1992, globally more than 100 patients with liver disease have been treated by HT (76–79). HT has been further developed with standardized techniques for hepatocyte isolation, culture, and storage. However, whenever hepatocytes are isolated or recovered, cell viability is often the only parameter to determine the quality of the material to be transplanted. In prior studies, cells with viability higher than 60% were used for HT based on trypan blue exclusion, but this test is not sufficient to determine hepatocyte functionality or to detect early apoptosis events. Standardized procedures to control hepatocyte viability and functionality as well as microbiological analysis before transplantation will be necessary for wider clinical applications and to improve patient outcomes (80).

Despite a number of encouraging reports about the clinical outcome of HT, the long-term efficacy of the treatment is only partially successful or transient, partly due to poor engraftment rates. Typically, 2–10% of the liver equivalent mass is injected (up to 100 × 10^6^ cells/kg); however, likely due to instant blood-mediated immune reaction or phagocytosis by immune cells activated by surface markers of injected hepatocytes, more than 70% of hepatocytes are rapidly lost within the first month of the treatment. HT with a high number of cells and multiple infusions can be performed, although 2 × 10^8^ hepatocytes per kilogram of body mass is considered an upper limit to prevent portal hypertension. The number of cells needed for transplantation also varies by the disease target or complication risk. For example, for patients with Crigler–Najjar syndrome, approximately 12% of the liver mass is necessary, whereas fewer cells can be used effectively for other metabolic disorders, such as glycogen storage disease 1a or ornithine transcarbamylase deficiency (81, 82). To improve engraftment rates, patients with HT require immunosuppressive regimens; however, there exists no consensus for immunosuppressive maintenance after HT. Moreover, there is no clear mechanism for immunosuppressive monitoring (i.e., OLT monitoring is completed by liver biopsy with standardized criteria and plans of action based on pathology findings; HT monitoring is less straightforward, as biopsy is insufficient to determine what is happening to dispersed cells throughout the hepatic parenchyma that are not as easily found or monitored). As a consequence, immunosuppressive regimens will require novel methods of cellular monitoring, which will require deeper studies to create a balance between the engrafted cells and host immune system in the patients’ livers (83–85).

Due to these constraints, the survival of cellular allografts drops over time likely because the transplanted hepatocytes do not necessarily have a selective advantage (and with cellular rejection by the immune system are more likely to be at a disadvantage) as compared to host hepatocytes (86, 87). Strategies to enhance the proliferative capacity of the engrafted cells, such as genetic modification of the donor cells, partial hepatectomy, irradiation, and partial portal vein embolization, have been actively researched in animal models (72). Combinations of these approaches were used whereby a transgene is coexpressed with a short hairpin RNA that gives modified hepatocytes resistance to a specific drug-induced hepatotoxicity enabling selection for the transplanted cells over time (88). Taken together along with emergent and time-sensitive clinical scenarios will often rely on hepatocytes isolated in a variety of conditions and qualities, many complementary approaches will be required to improve the long-term efficacy and outcome of HT.

### Fetal Hepatocytes

Since the first hepatocyte transplantation in 1992 (89), primary cells are the main cellular source for cell-based therapy to treat acute liver failure and inborn errors of metabolism. However, the worldwide shortage of donor organs also results in an insufficient source of liver tissue that can be used for high-quality hepatocyte isolation, which has led to the exploration of other alternative sources that can be used for cell-based therapy. Fetal and neonatal livers have been suggested as potential substitutes, although transcriptomic comparison revealed a significant difference between fetal and adult samples, as the expression of cytochrome P450 enzymes was higher in adult hepatocytes while fetal samples had more DNA replication and expression of repair-related genes (90). Animal studies have shown that fetal hepatocytes are capable of differentiating into mature hepatocytes after being intrasplenically injected to the liver, although engraftment is poor compared with primary human hepatocytes (91); nonetheless, fetal hepatocytes on their own express several key genes important for liver detoxification, and preliminary trials have shown a mild clinical improvement with fetal hepatocyte transplantation in acute liver failure patients (92–94). Further analysis is still required to fully determine the phenotype of cells after transplantation and determine the long-term benefits of such treatment given the complex ethical and sourcing issues that will remain.

### Immortalized Hepatocytes

Liver regeneration is a highly regulated process that allows hepatocytes to proliferate and replace damaged areas. The regeneration cascade is dependent on the ability of hepatocytes to transition from a quiescent state and reenter the cell cycle in response to the secretion of various growth factors and cytokines such as EGF, FGF, HGF, TGF-β, TNF-α, and IL-6 (95). However, isolated hepatocytes in vitro lose most of their proliferative capacity, probably due to missing molecular cues and an inadequate physiological milieu. As a result, an immortalizing strategy was proposed to override cell senescence in vitro and stimulate cell growth. The common immortalizing methods involve the overexpression of (*a*) viral oncogenes such as E1A/E1B (adenovirus), SV40 large T antigen (SV40) and E6/E7 genes (papillomavirus), (*b*) the catalytic subunit of telomerase reverse transcriptase (hTERT), (*c*) the constitutively active hepatocyte growth factor receptor (cMet), or (*d*) E6/E7 genes to upregulate the oncostatin M (OSM) receptor and enable OSM-dependent expansion (96, 97). To increase the safety of these methods, gene excision using Cre recombinase (floxed genes) and the usage of temperature-sensitive proteins were also examined to decrease the chance of malignancy (98). While hepatocyte immortalization has been studied for many years, it still does not offer a viable clinical solution since immortalized hepatocytes with sufficient hepatic-like properties have not been achieved yet.

### Stem Cells

Stem cell–based therapies have attracted much interest in the last two decades and present a promising strategy to promote liver regeneration, with a plethora of research and clinical studies already published. Many experiments have examined the potential of using various stem cell types, including embryonic, mesenchymal, and induced pluripotent stem cells (iPSCs) for the treatment of a variety of liver diseases. To date, 143 clinical trials involving usage of stem cells from diverse origins to treat different liver diseases have been conducted, including 33 that are still ongoing (http://www.clinicaltrials.gov) (Table 1).

### Bone Marrow Stem Cells

Bone marrow stem cells (BMSCs) comprise hematopoietic stem cells (HSCs), mesenchymal stem cells (MSCs), and endothelial progenitor cells. The first usage of bone marrow for cell therapy dates back to 1956, when hematopoietic recovery was identified after bone marrow injection to repair irradiation-induced damage (99–101). Direct involvement of BMSCs during liver regeneration was also suggested due to an increase of HSCs detected in the peripheral blood and in the liver after partial hepatectomy (102, 103), with reports suggesting that BMSCs transdifferentiate in the liver (104, 105) and follow-up reports that BMSC–hepatocyte cell fusion is the likely mechanism of action (106, 107). Inhibition of BMSC migration after liver resection in a mouse model significantly impairs hepatocyte proliferation and liver mass regeneration (108). As an example, van Poll et al. (109) have shown that systemic infusion of an MSC-conditioned medium led to a significant reduction of hepatocellular death and further prompted cell proliferation in a liver damage rat model. Other proposed mechanisms include modulation of the immune response in the liver (110), inhibition of fibrosis after injury (111), and promotion of angiogenesis (112). A complete understanding of the beneficial effects of BMSC-based therapy will require more research to explain the appropriate therapeutic window, cell dosage, longevity, and durability of the benefit and to delineate the mechanisms underlying BMSC impact on liver regeneration and fibrosis.

### Human Induced Pluripotent Stem Cells

iPSCs hold great promise for personalized regenerative medicine and present a potential hepatocyte source for liver cell therapy. iPSCs can be differentiated to all three germ layers (113) and efficiently differentiate into a variety of cell types, including hepatocyte-like cells (iHeps) (114–117). iPSC-derived iHeps in culture exhibit many morphologic and phenotypic characteristics of human hepatocytes: cuboidal morphology, albumin secretion, glycogen synthesis, urea production, and inducible cytochrome P450 activity (115–117). iHeps were shown to be a valuable tool not only as a cell reservoir but moreover as a platform for liver-related studies. Cells generated from patients with genetic metabolic diseases such as α_1_-antitrypsin deficiency, familial hypercholesterolemia, glycogen storage disease type 2, Wilson disease, Tangier disease, tyrosinemia type 1, and Alpers–Huttenlocher syndrome were shown to recapitulate key phenotypes and disease pathological features in vitro (118). These disease-specific hepatocyte-like cells offer the opportunity to study these diseases in human cells as opposed to animal models or in cell lines that do not faithfully recapitulate the disease as manifested in humans. As an example, Cayo et al. (119) performed a drug screen on iHeps generated from iPSCs carrying a mutation in LDLR (isolated from a familial hypercholesterolemia patient) to identify potential compounds to treat hypercholesterolemia. iHeps were also used for the study of host-pathogen interactions of multiple liver infectious agents including hepatitis B virus (120), hepatitis C virus (121), hepatitis E virus (122) and *Plasmodium* (123). Several groups have shown the potential of using iPSCs for liver transplantation. As an example, Takebe et al. (124, 125) reported functional transplantation and maturation of 3D vascularized iHeps (liver buds) obtained from the triculture of hepatic endoderm (iPSC-HE) with human MSCs and human umbilical vein endothelial cells.

Despite the remarkable progress and advances to expand the therapeutic potential of iPSC-derived hepatocyte-like cells over the last decade, several hurdles prevent them from being used in a clinical setting. The first and most critical aspect is the safety concern of using iPSCs for cell therapy. Preexisting and new mutations during and after reprogramming were reported in iPSC lines, raising concerns regarding their tumorigenic potential (126), with oxidative phosphorylation suggested as one of the factors contributing to de novo mutations (127). UV damage induced mutations in ~50% of iPSCs derived from skin fibroblasts; in addition, iPSC subclonal mutations not present in the parental line were also detected. While the majority of mutations were located in closed chromatin regions, mutations that occurred during iPSC reprogramming showed an increased correlation with active chromatin and altered gene expression (128). More rigorous research is required to determine the genomic stability of iPSCs and their safety in cell therapy usage. Another concern relates specifically to iHeps. While differentiation protocols have significantly improved with differentiation efficiency reaching more than 90%, iHeps phenotypically are more similar to fetal human hepatocytes rather than adult hepatocytes. Hence, further development toward terminal differentiation of iHeps may be required before they are suitable for clinical use.

## TISSUE ENGINEERING TO IMPROVE CELL AND TISSUE THERAPY FOR CHRONIC LIVER DISEASES

### Tissue Engineering and Cirrhosis

**Figure 3:**
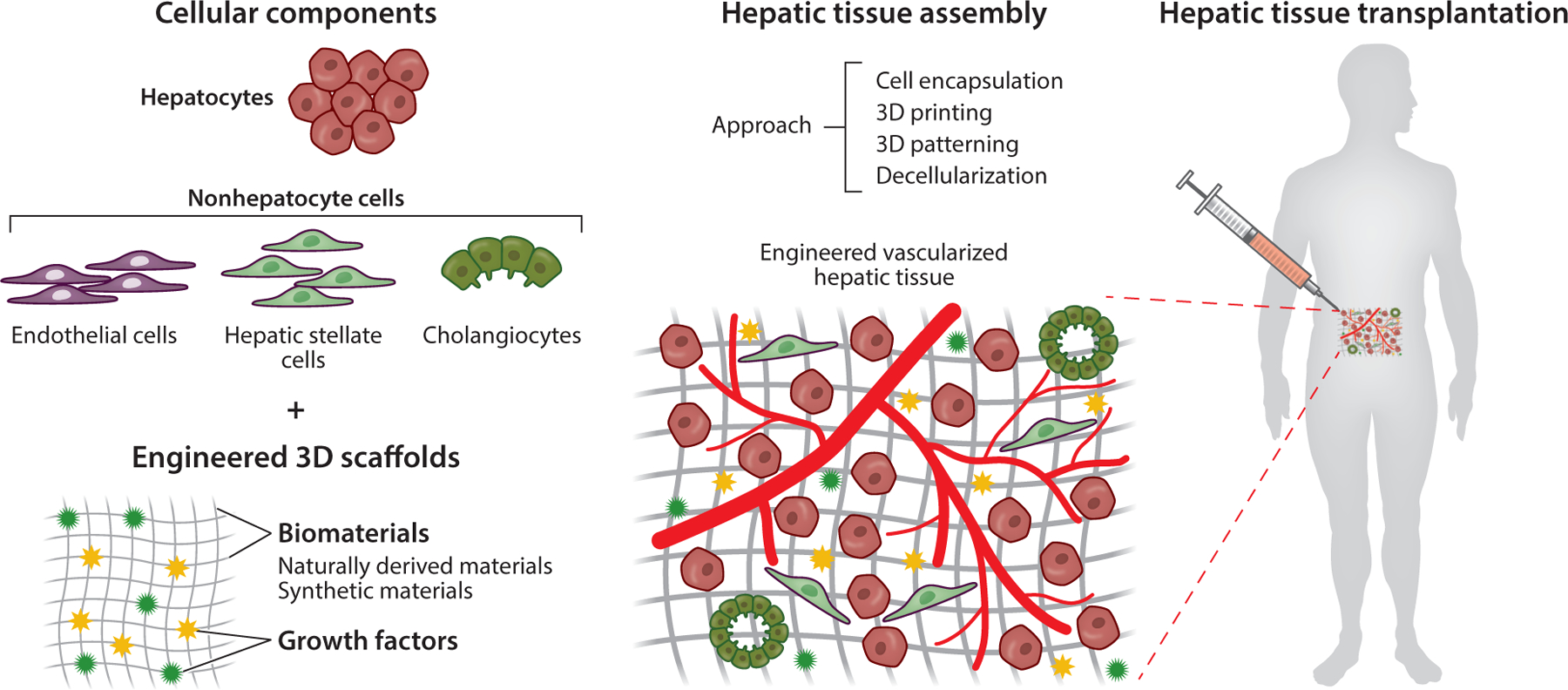
Liver tissue engineering approaches. Hepatic tissue engineering consists of cellular components and engineered 3D scaffolds that are assembled and transplanted in vivo to yield hepatic function.

Broadly defined, liver tissue engineering describes a construction of a functional hepatic tissue composed of biocompatible materials seeded with liver cells (hepatocytes, endothelial cells, hepatic stellate cells, etc.) with the ultimate goal to transplant the hepatic tissue grafts into patients with chronic liver diseases (Figure 3). A successful engineered liver tissue once implanted needs to integrate with the host vasculature to enable long-term survival of the implant. Important components that will require optimization to lead to a successful hepatic tissue graft include cellular sources, biomaterials, and implantation sites. We discuss each of these important components of an engineered hepatic tissue and we also discuss different approaches to construct an implantable engineered hepatic tissue. Although the ultimate goal is to engineer liver grafts to enhance or replace whole-organ transplantation, creation of functional hepatic tissue for in vitro models is also an attractive avenue to contribute to the understanding of liver disease progression and prevention. We also elaborate on tissue engineering platforms to model liver cirrhosis.

### Cellular Sources

As hepatocytes are the principle parenchymal cell type in the liver, optimization of hepatocyte sourcing and isolation is key for any liver tissue–engineering study (Figure 2). One key hurdle addressed earlier is that although hepatocytes possess a remarkable proliferative capacity in vivo, isolated hepatocytes gradually lose their functions and their regenerative capacity in vitro. Thus, their use in tissue engineering applications will require a large number of hepatocytes, and their use in implantable hepatic tissue has remained limited. Efforts to find an alternative source of primary human hepatocytes have led to studies using alternative cell types and/or approaches similar to that described for cell transplantation and include fetal hepatocytes, transdifferentiation of pancreatic cells into hepatocyte-like cells, direct reprogramming of fibroblasts into hepatocyte-like cells, and differentiation of pluripotent stem cells (PSCs) into iHeps (Figure 2). However, several hurdles still remain to successfully differentiate other cell lineages into mature hepatocytes before they can be used in clinical settings. An alternative and promising approach is to expand human hepatocytes in humanized animal models as almost human liver incubators or bioreactors. Currently, humanized mice have been generated to host and expand primary human hepatocytes (129). Future development on humanized large animals might be a step forward to permit sufficiently large-scale expansion of primary human hepatocytes for clinical applications.

Though such efforts in hepatic tissue engineering have focused mainly on hepatocytes, the liver is also composed of nonparenchymal cells such as LSECs, hepatic stellate cells, Kupffer cells, and cholangiocytes (Figure 3). Each of these cell types contributes specifically to the global functions of the liver. For example, Kupffer cells are the major phagocytic cells that safeguard the liver by engulfing pathogens through the portal and arterial circulation. Activated hepatic stellate cells (believed to be the principal cell type contributing to fibrosis formation) are also important in the liver regenerative process. Similarly, LSECs serve as a channel for fluidic transport and help to modulate vascular tone in the liver, but they also function by secreting angiocrine growth factors to promote liver regeneration. They are also thought of as gatekeepers to regulate inflammation in the liver by restricting or enabling entry of leukocytes from the bloodstream. To successfully engineer a liver graft, besides including hepatocytes as a major cellular source, it may be important to incorporate other cell types into the engineered hepatic tissue to fully recapitulate the physiological functions of the liver. Though some of the nonparenchymal cells show much more proliferative potential in vitro such as liver endothelial cells and hepatic stellate cells, the hepatic graft could potentially require a large quantity of such cells, which might not be feasible to acquire through in vitro expansion. Whether the inclusion of nonparenchymal cells in engineered hepatic tissue is beneficial for graft function and transplantation or triggers the host immune system to reject the tissue graft remains largely unknown. As a result, other strategies to use patient-derived iPSCs to differentiate into different liver nonparenchymal cells or directly reprogram patient-derived fibroblasts into liver nonparenchymal cells might be promising alternatives to solve the shortage of liver nonparenchymal cells or to overcome host immune rejection.

### Biomaterials

Unlike direct cell-injection therapies, engineering implantable hepatic tissues relies on the incorporation of cells into 3D scaffolds made from biomaterials that are compatible with the host. These scaffolds serve as structural and biological supports for the hepatic cells in several contexts: promoting cellular attachment and organization, enhancing survival of the embedded hepatic cells, and facilitating integration to the host vasculature to enable long-term survival of the implanted scaffolds (Figure 3). The main classes of biomaterials that have been used in hepatic tissue engineering include synthetic biomaterials, naturally derived polymers, and decellularized matrices. In addition to the ability to control the structure of the scaffolds, biomaterials’ mechanical properties can be optimized to define pore sizes, stiffness, and geometry to better support hepatic cells. Additionally, biomaterials can also be modified and tethered with bioactive and controlled-release signaling growth factors to promote hepatic functions. Regardless of the progress in designing biomaterials for tissue engineering in general, the majority of studies using biomaterials in liver tissue engineering have been done in vitro. Perhaps, given the complexity of the liver as a multifunctional organ, there are several challenges and parameters that require optimization in the biomaterial scaffolds in vitro before they can be applied in in vivo applications and further in clinical settings.

### Synthetic Materials

One of the advantages of using synthetic biomaterials is the ability to synthetically design and easily tune various parameters such as stiffness, biodegradability, and porosity. For example, some common synthetic materials for liver engineering include poly(vinyl alcohol) (PVA), poly(glycolic acid) (PGA), poly(lactic acid) (PLA), copolymer blends of PLA and PGA (PLGA), and poly-l-lactic acid (PLLA). PVA is nonbiodegradable while PGA, PLA, and PLGA are controllable biodegradable materials. Scaffolds made from PLGA seeded with hepatocytes and liver nonparenchymal cells were used in vitro in both static and continuous flow conditions to demonstrate that embedded hepatic cells attach, survive, and synthesize albumin in these scaffolds (130). Another commonly used biomaterial in tissue engineering application is polyethylene glycol (PEG). For example, primary human hepatocytes encapsulated inside 3D PEG hydrogels, coupled with arginylglycylaspartic acid (RGD) peptide, sustained albumin secretion and urea synthesis for up to eight days in vitro. Some human albumin was detected in circulation once these hepatocyte-encapsulated PEG hydrogels were implanted into the peritoneal cavity of athymic nude mice, suggesting the survival of primary human hepatocytes in the PEG hydrogel implants (131). PEG was also coupled to hyaluronan for culturing iPSC-derived hepatocytes in a microfluidic platform and was shown to increase cell viability and albumin secretion as compared to agarose or alginate (132).

In addition to the in vitro studies, some of these synthetic biomaterials have also been used for animal studies. For example, Jiang et al. (133) used PLLA scaffold first seeded with fetal mouse liver cells and cultured in vitro in medium supplemented with HGF, FGF1, FGF4, and oncostatin-M. Subsequent implantation of these PLLA scaffolds in the peritoneal cavity of 70% hepatectomized mice showed a higher presence of albumin-positive engrafted cells 15 days after transplantation (133). Additionally, porous scaffolds of PVA or various PLGA blends with hepatocytes were transplanted to the small intestinal mesentery of rats and sustained the survival of hepatocytes up to 6 months (134).

### Naturally Derived Biomaterials

Besides synthetic biomaterials, biomaterials derived from natural components have also been utilized in liver tissue engineering. Some common naturally derived materials include alginate, chitosan, fibrin, collagen, and Matrigel. Alginate, a natural polysaccharide, has been used to encapsulate hepatic cells in an extracorporeal perfusion system, while galactosylated chitosan was made into nanofibers with electrospinning to form 3D scaffolds that support a higher level of albumin secretion, urea synthesis, and cytochrome P450 enzyme expression in rat hepatocytes (135). Another material, Matrigel, is composed of a mixture of several basement membrane matrices such as laminin, entactin, and collagen IV. Laser-guided direct writing was able to micropattern the self-assembly of hepatocytes and endothelial cells to form liver sinusoid-like structures with long-term function on Matrigel (136). Recent advances in culturing hepatic organoids using 3D Matrigel in vitro with TNFα have led to the expansion of hepatic cells (137). These in vitro expanded hepatic cells, once implanted in mice with injured liver, could repopulate the mouse liver to rescue the liver function. Collagen I has also been widely used to culture hepatocytes. Primary hepatocyte functions such as albumin secretion, cytochrome P450 activity, urea synthesis, and bile acid homeostasis are greatly enhanced and stable up to 14 days in vitro when hepatocytes are sandwiched between layers of collagen I hydrogel (138). Thus, the collagen sandwich assay has become a useful in vitro platform to perform drug screens and toxicity studies for hepatocytes.

Fibrin gels are perhaps also one of the most widely used natural hydrogels for both in vitro and in vivo hepatocyte implantation. Fibrinogen proteins once cleaved by thrombin rapidly assemble to form a network of fibrin gel. Because fibrinogen is secreted by hepatocytes, fibrin gel is biocompatible with hepatocyte cultures. Fibrin gel has also been shown to increase proliferation of human fetal liver progenitor in coculture with endothelial cells (139). Recent work by Stevens et al. (140) demonstrated that human hepatocytes sustained albumin secretion up to 80 days after implantation in immune deficient mice when they were embedded as aggregates of hepatocytes and fibroblasts together with patterned endothelial cells within fibrin gels. These hepatic fibrin constructs also showed an elevated level of albumin in response to liver injury in the mouse model (140).

Liver tissue nevertheless does not contain one specific type of extracellular matrix (ECM) but rather a mixture of organ-specific matrices. Hence, to mimic the liver microenvironment that best supports hepatocyte function, a hydrogel made from decellularized liver ECM might be an alternative biomaterial for liver tissue engineering applications. Some advantages of a liver-specific matrix hydrogel are the solubilized liver-specific matrices that could maintain the physical properties of the liver ECM in vivo, the composition of different ECMs, the stiffness of the ECMs, the porosity of the ECMs, or the liver-specific growth factors tethered to the liver ECMs. For example, porcine liver ECMs were solubilized and made into a hydrogel for primary rat hepatocyte cultures in vitro. The rat hepatocytes survived and sustained their hepatic functions for up to three weeks in culture (141).

### Implantation Sites

The classic hepatocyte transplantation model involves delivery of the hepatocytes into the portal vein. Once entering the bloodstream of the portal vein, a fraction of transplanted hepatocytes could cross the endothelium to enter the space of Disse and gradually populate the injured liver. This transplantation method is a simple procedure and could be carried out in severely ill patients as compared to more complicated orthotopic transplantation of hepatocytes. Early studies of hepatocyte transplantation suggested that the transplantation site also dictates the function of hepatocytes. For example, an early study demonstrated improvement in hepatocyte function after transplantation in a synthetic biodegradable polymer scaffold with direct access to the portal venous system. This study led to a notion that transplanted hepatocytes might function better when they are exposed to the hepatotropic factors present in the portal vein (141a). However, in cases of severely ill patients with elevated portal venous pressure, surgical procedures involving the portal vein are not feasible and will further complicate the patient’s illness. Hence, other surgical sites have further been explored as alternatives. One of the key features of a suitable implantation site is its ability to enable implanted constructs or cells to get access quickly to the host vasculature for long-term survival of the implanted constructs. For example, in a mouse model with reproducible liver injury, a highly vascularized lymph node has been shown to be a supportive environment for transplanted hepatocytes (142). Other studies also showed that peritoneum such as the mesenteric fat pad or renal capsule are also appropriate sites for liver tissue engineering, as these areas are highly vascularized and enable integration of the engineered tissue to the host environment (143). From these studies, it is important to draw a conclusion that ectopic transplantation for engineered liver tissues is possible to maintain and support transplanted hepatocytes in vivo. However, it remains to be determined whether bypassing the portal vein access of the engineered liver tissues would have an effect on some of the physiological functions of the liver, which are to process and metabolize the nutrient-enriched blood before the blood is returned to the systemic circulation.

Besides the requirement for easy access to highly angiogenic ectopic transplantation sites, another critical parameter that must be considered for ectopic transplantation is the need for sufficient space at the ectopic sites to host a large number of transplanted cells (144). The kidney capsule and lymph node might not be ideal transplantation sites due to the limited space for large liver tissues. Intraperitoneal sites might provide sufficient space and a vascularized network to support the liver tissue. However, internal ectopic transplantation sites impose challenges on efforts to monitor the transplanted sites. On the other hand, the subcutaneous space requires less invasive surgical procedures for both transplantation and monitoring, but it is a less vascularized tissue (145). Subcutaneous implantation also poses aesthetic challenges when the implanted tissues protrude out of the patient’s skin. There might not be an ideal implantation site; rather, future efforts should focus on incorporating advances in designing engineered scaffolding and biomaterials that directly enhance angiogenesis at the implantation sites. These approaches might open more possibilities for implantation in different ectopic sites that fit the therapeutic needs of the patients.

## STRATEGIES FOR DEVELOPING ENGINEERED LIVER TISSUES

### Encapsulating Cells in Scaffolds

One of the most commonly used methods to fabricate engineered liver tissue is to encapsulate cells in 3D biomaterials (Figure 3). This method has been used both in vitro to study hepatocyte biology and in in vivo settings for implantation. With this method, the construction of the engineered liver graft is fairly straightforward. Scaffolds can be easily constructed by simply re-suspending a desired number of hepatocytes or spheroids of hepatocytes inside a biomaterial of choice. Besides hepatocytes, other nonparenchymal cells such as liver-derived endothelial cells or stellate cells could also be intermingled into the scaffolds. The scaffold can then be implanted into the animal models for further studies. For example, Cima et al. (146) pioneered using synthetic scaffolds to examine functions of hepatocytes in vitro and found that PLGA was supportive of hepatocyte function in vitro. Subsequent work by Mooney et al. (147) used a PLGA scaffold with encapsulated hepatocytes and implanted it into mice and showed engraftment of the liver scaffold. Other groups also generated alginate microbeads to encapsulate primary human hepatocytes and subsequently transplanted them into the intraperitoneal site in animal models. These encapsulated hepatocytes inside alginate carriers improved the liver function of mice with acute liver failure (148). Similarly, iPSC-derived hepatocyte-like cells were aggregated into spheroids and encapsulated into alginate capsules and implanted into the intraperitoneal cavity. After 24 days in vivo, the capsules of iHeps showed improved albumin and urea secretion and cytochrome P450 activities as compared to standard culture in 2D before implantation (91).

### 3D Fabrication for Engineered Liver Scaffold

Though cell encapsulation within biomaterial scaffolds is a simple method to generate implantable engineered liver tissues, most of the studies using cell encapsulation often share a similar observation: The number of hepatocytes diminish in the implanted liver tissues, especially during the initial phase of implantation. It is possible that the implanted liver tissues are not well integrated with the host vasculature and rely on pure diffusion of nutrients into the scaffold to support survival of the implanted hepatocytes (149). Tissue engineering efforts need to be directed toward improving the integration of the engineered liver tissues into the host vasculature. Several strategies can be used to address this shortcoming. One strategy is to design biomaterials that promote integration with the host vasculature by altering the pore sizes and mechanical properties of the biomaterials. Alternatively, biomaterials can be tethered with angiogenic factors to stimulate angiogenesis and invasion of the host endothelial cells into the scaffolds (150).

Although altering the physical and biomechanical properties of the scaffolds could improve the host vasculature invasion, vascularization of the implanted liver construct remains a slow process and might not be rapid enough to vascularize the whole implant to support the survival of the implanted cells (or for that matter the patient). Another approach is to vascularize the engineered liver tissues before implantation by either embedding endothelial cells or building vascular structures within the engineered liver tissues. This strategy will now depend on the ability of the engineered vascularized liver tissues to anastomose to the host vasculature. For example, Baranski et al. (151) used photolithography technology and molding to build cord-like structures of endothelial and stromal cells. Once these endothelial/stromal cords were implanted, they quickly anastomosed to the mouse vasculature and were perfused after three days. A similar approach to coimplant endothelial cell cords with primary human hepatocyte aggregates in engineered liver tissues into mesenteric fat pad also showed positive results. Endothelial cell cords co-implanted with human hepatocyte aggregates outperformed conditions where single endothelial cells were coimplanted with primary human hepatocyte aggregates (151). This work supports the beneficial strategy to implant prevascularized liver tissues. A more complex and advanced approach to build a vascularized network within the engineered tissues is to use 3D printing technology. With this technique, a more defined and multilayer vasculature network and complicated geometry can be constructed. Recent work by Grigoryan et al. (152) showed that a complex geometry of vasculature can be built and deployed for transplantation with primary human hepatocytes to support hepatocyte function in a chronic liver injury mouse model.

### Decellularization for Engineered Liver Scaffold

Alternatively, bottom-up engineering approaches to build and pattern vasculature and liver architecture using lithography microfabrication, 3D molding, or 3D printing in conjunction with biomaterials have shown some promising results to advance our current method of liver transplantation and improve the design of liver engineering scaffolds (153). However, the liver is a complex biological system with a well-defined architecture that is specified by zonation across the central vein to portal vein and possibly unique liver-specific extracellular matrices or active liver-derived biochemical proteins embedded within the liver tissues. These features will certainly make it challenging to accurately engineer liver tissues with a bottom-up approach. To circumvent the obstacles to engineer the liver tissue architecture from scratch, an alternative approach has taken advantage of the preserved physiological organ features (vasculature network, ECM components, architecture) by decellularizing the cellular components of an organ, leaving behind the intact ECM scaffold. This novel approach was pioneered by Ott et al. (154) in 2008 to engineer bioartificial hearts. After removing the cellular components of the rat heart with detergent via perfusion in the coronary arteries, the authors demonstrated that endothelial cells and cardiac cells could be reintroduced back into the rat heart scaffolds and kept alive under perfusion ex vivo for 28 days (154). Following a similar approach but using a rat liver, Uygun et al. decellularized rat livers through perfusion at the portal vein to obtain the intact liver matrix scaffold, including the vascular bed. Upon decellularization of the liver, the authors reintroduced hepatocytes and endothelial cells into the liver graft. The liver graft was cultured in vitro for 8 days with fewer than 20% apoptotic cells detected. To demonstrate the feasibility of this method, the authors transplanted the liver graft via a donor renal vein and artery and performed an 8-h in vivo perfusion. After 8-h perfusion, the graft exhibited minimal damage as compared to before in vivo transplantation (155).

### Animal Models of Hepatocyte Cell Transplantation

The basis for clinical use of cell therapy currently depends on the demonstration of efficacy in animal models of acute and chronic liver diseases. Experimental animal models are crucial for understanding the underlying pathophysiology and the molecular mechanisms involved in liver disease progression and resolution. Animals frequently used to study liver-associated metabolic disorders are shown in Table 2 (see also 156–161).

Small animal models including mouse and rat have been studied for more than 100 years. There is more known about rodent genetics and biology than any other experimental animal, and the protein coding regions are similar to those of humans (162). These animals are of great interest to study liver disease because they are genetically resistant to successive breeding and have a short gestational period and life span; thus, they can generate experimental analyses more quickly. Small animal models are relatively inexpensive, reproduce quickly, and are easier to manipulate genetically compared with larger animal models. Although these models are extensively used, mice have a dissimilar immune system, are insulin resistant, and have faster metabolic rates than humans do. These differences contribute to recurrent failures in translating clinical therapies from small animals into humans.

The failure of small animals to precisely recapitulate human liver disease phenotypes has driven research toward animal species that more closely resemble humans in terms of immune system, physiology, and behavior. Large animal models such as rabbits, pigs, dogs, and nonhuman primates more closely resemble human conditions than do small animal models. Their large size allows for easier drug dosing and repetitive blood sampling. This is necessary to clinically translate relevant experimental findings, and their longer life span allows longitudinal studies. Compared with both small and large animal models, nonhuman primates are most closely related to humans in liver anatomy and hepatic vascularization. However, these animal models are accompanied by several ethical concerns and difficulties in husbandry, incur higher costs for specialized care, and pose an increased risk for transmitting viral diseases.

Despite the multiple drawbacks with using small and large animal models to study human liver diseases, scientists continue to use animals in combination with embryonic stem cells, organ-derived stem/progenitor cells, and circulating stem/progenitor cells to improve liver cell transplantation therapies, so that such therapies can be translated into the clinic. In Table 2, we list the most commonly used model systems for hepatocyte cell transplantation.

### Modeling Cirrhosis Using Liver Tissue Engineering

As we have discussed in sections above, cirrhosis is the end stage of liver fibrosis. Despite the remarkable regenerative capacity of the liver, continuous insults to the liver can damage the hepatic capacity to regenerate, generate inflammation, and gradually lead to accumulation of scar tissue. In several cases of liver fibrosis, one of the most common drivers of this disease is hepatocyte cell death. Damaged hepatocytes release reactive oxygen species, recruit inflammatory white blood cells, and stimulate the fibrogenic response of hepatic stellate cells. Inflammatory cells promote activation of hepatic stellate cells to synthesize and deposit excessive ECM proteins and secrete a plethora of factors that can in turn reinforce further recruitment of inflammatory cells (163). LSECs can also be maladaptive and serve as a profibrogenic niche to enforce proliferation of activated hepatic stellate cells (164). Therefore, a vicious circle where profibrogenic cells activate and stimulate one another is likely to occur in chronic liver fibrosis. Without sufficient intervention, the excessive deposition of ECMs gradually leads to destruction of the liver architecture, obstruction of blood flow, and cirrhosis. Current approaches to understand liver fibrosis and cirrhosis have relied mostly on rodents such as the transgenic MDR2 knockout mice, DDC treatment, choline-deficient ethionine-supplemented chow, bile duct ligation, and CCL4-induced liver injury (165). These models have allowed us to gain valuable insights into the biology of liver fibrosis and cirrhosis. However, in addition to the low-throughput experimental setup of animal models and the complexity of this disease, it is also challenging to perform very detailed mechanistic studies. Moreover, animal models do not always pathologically recapitulate human liver diseases.

Consequently, liver tissue engineering models using human cells can serve as complementary platforms to model liver diseases, especially cirrhosis. A biomimetic in vitro liver tissue engineering model will ideally include several cell types that partake in the fibrogenic cascade such as hepatocytes, hepatic stellate cells, LSECs, and immune cells. However, liver tissue engineering models also have to overcome many hurdles. One of the most important challenges for liver tissue engineering models is to maintain physiological functions in vitro. For example, hepatocytes, one of the key players in liver fibrosis, quickly lose their physiological functions in in vitro culture. Additionally, hepatic stellate cells once isolated and cultured in 2D culture often acquire an activated phenotype. Similarly, LSECs quickly lose their typical fenestra once cultured and expanded in 2D culture.

Some elegant 2D studies using rat HSCs on polyacrylamide gels showed that hepatic stellate cells transformed into myofibroblasts on stiff substrates but remained quiescent on soft substrate (166). However, traditional 2D culture might not mimic hepatic cells in vivo. As such, 3D models of liver tissues have provided an additional complexity to the systems. One of the most popular 3D cultures for liver tissues is formed by liver spheroids using a low-attachment 96-well plate or hanging drop method. The hepatic spheroids can include hepatocytes with hepatic stellate cells, endothelial cells, and Kupffer cells. For example, Leite et al. (167) generated spheroids of HepaRG (hepatocyte cell line) and passaged primary hepatic stellate cells and demonstrated that hepatic stellate cells reverted to a quiescent state. However, once challenged repeatedly with allyl alcohol and methotrexate, HSCs reactivated by upregulating their activated markers such as COL1A1, and LOXL2 (167).

To further provide additional complexity to in vitro models of liver fibrosis, microfluidic-based liver models have also been developed to incorporate shear forces into the coculture system. For example, primary hepatocytes were cocultured in a transwell device with HSCs, and macrophages in a perfused medium supplemented with high glucose, insulin, and free fatty acid, which are known to cause nonalcoholic steatohepatitis liver injury. After 10 days of exposure, alpha smooth muscle (αSMA)-positive cells increased but hepatic stellate cells showed no deposition of collagen, suggesting some incomplete activation of hepatic stellate cells in response to the liver injury factors (168).

Despite the improvement in in vitro models of engineered liver tissue, these models are still limited and so far have not fully recapitulated liver fibrosis, in part because of the complexity of the disease. Additionally, most of these models still lack some important features of the liver such as inclusion of 3D sinusoidal vascular network, cholangiocyte ductal network, or zonation of the liver, and more importantly maintenance of the proper regenerative capacity and response to injury of hepatocytes (169).

## CONCLUSIONS

Liver disease is a growing and important clinical problem, affecting about 10% of the US population, and is the 11th-leading cause of death in the United States. The increasing population of patients with liver disease has resulted in more patients with end-stage liver disease or hepatocellular carcinoma, increasing the need for liver transplantation. In this review, we have examined the state-of-the-art approaches for cell- and tissue-based therapies for chronic liver disease while dissecting the mechanisms of liver injury, repair, and regeneration. We discussed several areas where our incomplete understanding has hampered our ability to move cell- and tissue-based therapies for liver disease forward into the clinical setting.

The use of cellular transplantation, while promising, has unfortunately been more challenging to implement in practice. Due to a variety of technical issues (e.g., cell sourcing, maintenance, viability) and clinical issues (e.g., cell dosing, implantation, monitoring of cellular rejection), cell transplantation has not yet lived up to its full potential. Addressing these different technical and clinical issues will be difficult. However, improvements in potential alternatives for cell sourcing (stem cell–derived hepatocytes or cell-cultured hepatocytes) and noninvasive tools for organ rejection monitoring have increased the likelihood of rapid improvements and potential clinical implementation in the near future. Improved understanding of the cellular and microenvironmental underpinnings of the hepatic microenvironment and development of the materials used along with optimization of their biophysical properties such as stiffness, biodegradability, and porosity for hepatocytes specifically and the liver in general and cellular organization have resulted in the generation of mini-livers that replicate various components of hepatic function. Insights into the appropriate biomaterial composition, ideal implantation sites, and tissue vascularization still require further improvement. Alternatively, to circumvent the challenges to engineering the liver tissue architecture de novo, organ decellularization maintains a variety of organ features including the architecture, the vascular network, and the spatial organization of chemical ligands. While this is promising, recellularizing these organ scaffolds has remained a challenge. Overall the community has made great progress in a variety of different domains to address the distinctive challenges that exist for the varying cell- and tissue-based therapies, presenting an exciting future for the field.

## Figures and Tables

**Table 1:** Clinical trials involving usage of stem cells from diverse origins to treat different liver diseases

Cell types incorporated	Trial status	Clinical trials reference
Autologous bone marrow MSCs for liver cirrhosis	Recruiting	NCT03626090
Autologous CD133 endothelial progenitor cells for liver cirrhosis	Recruiting	NCT03109236
Skin-derived ABCB5-positive MSCs for acute-on-chronic liver failure	Recruiting	NCT03860155
Human umbilical cord MSCs for decompensated HBV cirrhosis	Recruiting	NCT03826433
Umbilical cord–derived MSCs for decompensated liver cirrhosis	Recruiting	NCT03626090
Cellgram^™^ (bone marrow–derived MSCs)	Recruiting	NCT03838250
Allogeneic umbilical cord MSCs for liver cirrhosis caused by HBV	Recruiting	NCT04357600
Umbilical cord–derived MSCs for biliary atresia	Recruiting	NCT04522869
MSCs and T regulatory cells for decompensated cirrhosis	Not yet recruiting	NCT03460795
MSCs for acute-on-chronic liver failure	Not yet recruiting	NCT03863002
Stem cells from human exfoliated deciduous teeth for HBV-related liver cirrhosis	Not yet recruiting	NCT03957655
Bone marrow mononuclear stem cells for children suffering from liver cirrhosis due to biliary atresia	Recruiting	NCT03468699
Biomarker analysis of sinusoidal obstruction syndrome	Active, not recruiting	NCT03132337
Allogenic hematopoietic stem cells for HBV immunity	Enrolling by invitation	NCT03511794

Abbreviations: HBV, hepatitis B virus; MSC, mesenchymal stem cell.

**Table 2:** Animal models used frequently to study liver-associated metabolic disorders

Animal model	Corresponding human disease	Reference
Fumarylacetoacetate hydrolase^–/–^ knockout mouse	Tyrosinemia type I	140
mdr2 mouse	Progressive familial intrahepatic cholestasis type 3	156
spf-ash mouse	Congenital ornithine transcarbamylase deficiency	157
Long Evans Cinnamon rat	Wilson disease	158
Gunn rat	Crigler–Najjar syndrome type I	159
Watanabe heritable hyperlipidemic rabbit	Model for low-density lipoprotein receptor deficiency	160
Hyperuricemic Dalmatian dog	Hepatocyte transplantation for hyperuricemia disorders	161
